# Transmission Dynamics of Highly Pathogenic Avian Influenza Virus A(H5Nx) Clade 2.3.4.4, North America, 2014–2015

**DOI:** 10.3201/eid2410.171891

**Published:** 2018-10

**Authors:** Dong-Hun Lee, Mia Kim Torchetti, Joseph Hicks, Mary Lea Killian, Justin Bahl, Mary Pantin-Jackwood, David E. Swayne

**Affiliations:** US Department of Agriculture, Athens, Georgia, USA (D.-H. Lee, M. Pantin-Jackwood, D.E. Swayne);; US Department of Agriculture, Ames, Iowa, USA (M.K. Torchetti, M.L. Killian);; University of Texas School of Public Health, Houston, Texas, USA (J. Hicks, J. Bahl)

**Keywords:** highly pathogenic avian influenza virus, avian influenza, transmission, epidemiology, Eurasian H5, clade 2.3.4.4, United States, USA, North America, Canada, H5N2, H5N8, commercial poultry, chicken, turkey, wild birds, Midwest, Pacific flyway, Mississippi flyway, surveillance, viruses, respiratory infections, zoonoses

## Abstract

Eurasia highly pathogenic avian influenza virus (HPAIV) H5 clade 2.3.4.4 emerged in North America at the end of 2014 and caused outbreaks affecting >50 million poultry in the United States before eradication in June 2015. We investigated the underlying ecologic and epidemiologic processes associated with this viral spread by performing a comparative genomic study using 268 full-length genome sequences and data from outbreak investigations. Reassortant HPAIV H5N2 circulated in wild birds along the Pacific flyway before several spillover events transmitting the virus to poultry farms. Our analysis suggests that >3 separate introductions of HPAIV H5N2 into Midwest states occurred during March–June 2015; transmission to Midwest poultry farms from Pacific wild birds occurred ≈1.7–2.4 months before detection. Once established in poultry, the virus rapidly spread between turkey and chicken farms in neighboring states. Enhanced biosecurity is required to prevent the introduction and dissemination of HPAIV across the poultry industry.

Highly pathogenic avian influenza virus (HPAIV) A(H5N1) emerged in 1996 in Guangdong, China (A/goose/Guangdong/1/1996 [Gs/GD]), and has since caused outbreaks in poultry, infections in wild birds, and often fatal clinical cases in humans in >80 countries (*1*). In late autumn 2014, Gs/GD H5N8 clade 2.3.4.4 was reported in East Asia, Europe, and the west coast of North America (*2*,*3*). The timing and direction of this virus’s dissemination, which coincided with waterfowl migration patterns, together with phylogenetic and geospatial data (*3*,*4*) support the hypothesis that Gs/GD H5N8 clade 2.3.4.4 was introduced into North America through the Beringian Crucible via intercontinental movement of migratory waterfowl (*5*,*6*).

In November and December 2014, novel reassortant H5N1, H5N2, and H5N8 viruses were detected in wild waterfowl along the Pacific flyway (*2*,*7*). Reassortant H5N2 virus was identified as the causative agent of influenza outbreaks in poultry farms in British Columbia, Canada, and among wild waterfowl in the northwestern United States (Figure 4) (*8*). This virus subsequently was the predominant strain during HPAIV outbreaks among poultry in the United States in 2015, particularly during March–June (*9*,*10*), and was detected in wild birds migrating along the Pacific, Central, and Mississippi flyways (*11*,*12*). The last reported virus detection related to the outbreak in the United States was in June 2015 from a Canada goose in Michigan. Despite increased surveillance efforts in the United States, HPAIV was rarely detected in wild bird populations during the latter half of 2015 and 2016 (*13*). In 2015, the virus was detected just 2 times by real-time reverse transcription PCR in mallards, once in July (from bird banding efforts in Utah) and once in November (from a hunter harvest in Oregon) (*14*). In 2016, H5N2 virus clade 2.3.4.4 was detected in 2 mallards sampled in Alaska and Montana, providing evidence for low-level maintenance of this virus in wild birds in northwestern North America (*15*,*16*). Despite disagreement about the role of flyways in limiting viral spread among wild birds (*17*,*18*), the rapid spatial diffusion and transmission of HPAIV in wild and domestic birds highlight the need to further investigate the processes involved in viral emergence and spread.

**Figure 4:**
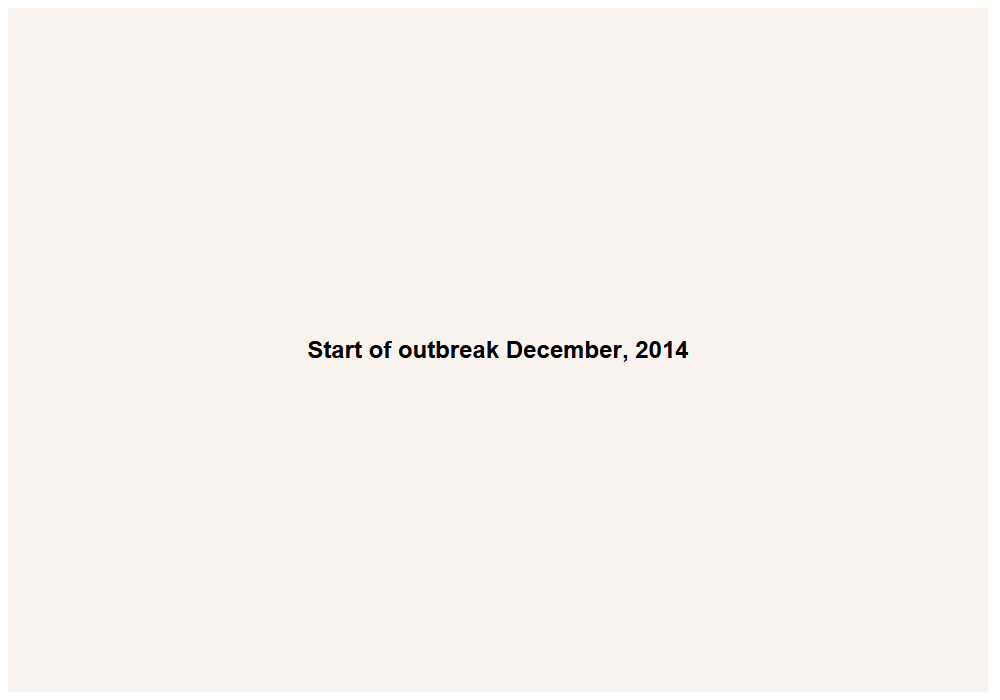
Outbreak of H5N2 influenza virus in birds of backyard and industrial farms, raptors, and wild birds in the United States and Canada, December 2014–July 2015. Despite increased surveillance throughout 2015 and 2016, few cases were reported after the outbreak. In 2016, a wild bird in Alaska was positive for H5N2, indicating that low-level transmission of this virus had occurred without being detected.

We sequenced and analyzed the full-length genome sequences of 249 H5N2 and 19 H5N8 HPAIVs collected during the 2014–2015 outbreaks in the United States. Viruses came from 32 wild birds, 7 raptors, 14 backyard poultry farms, and 196 commercial poultry sites. We used a molecular epidemiologic approach involving genome sequences and outbreak information to determine the evolution and spread patterns of these viruses.

## Materials and Methods

### Genome Sequencing

The National Animal Health Laboratory Network conducts PCR testing for avian influenza in the United States, and per title 9, Code of Federal Regulations, nonnegative samples are forwarded to the National Veterinary Services Laboratories (Ames, Iowa, USA) for confirmation testing and genome sequencing. In December 2014, interagency wild bird surveillance started including testing for the HPAIV Gs/GD lineage in wild birds in the Pacific flyway, and by summer 2015, surveillance was further expanded to include testing for this virus in all flyways (*19*). In our study, we included all available wild bird (n = 32) and raptor (n = 7) viruses from these surveillance efforts and, for poultry, just 1 virus from each affected site with available samples (n = 210; 14/21 affected backyard sites, 196/211 affected commercial flocks) (*20*).

We extracted viral RNA from 249 H5N2-positive and 19 H5N8-positive oral or cloacal swab samples using MagMAX Viral RNA Isolation Kit (Ambion, Foster City, CA, USA). We synthesized complementary DNA and amplified all 8 segments by reverse transcription PCR using SuperScript III (Invitrogen, Carlsbad, CA, USA) (*21*). We conducted genome sequencing using the Ion Torrent PGM (Life Technologies, Waltham, MA, USA) or MiSeq (Illumina, San Diego, CA, USA) platforms as previously described (*7*,*16*). We assembled genome sequences using SeqMan NGen version 4 (http://www.dnastar.com/t-nextgen-seqman-ngen.aspx). We deposited nucleotide sequences in GenBank (Technical Appendix Table 5).

### Maximum-Likelihood Phylogenetic Analysis of US HPAIV H5N2 

Of the 265 H5N2 sequences analyzed, we retrieved the 16 viruses from Canada from the GISAID EpiFlu database (http://platform.gisaid.org) and sequenced the 249 viruses from the United States. In all, 32 viruses were from wild waterfowl in 10 states (Alaska, Idaho, Kansas, Kentucky, Michigan, Missouri, Montana, Oregon, Washington, Wyoming); 7 viruses were from captive and wild raptors in 5 states (Idaho, Minnesota, Missouri, Washington, Wisconsin); and 210 viruses were from backyard and commercial poultry farms in 13 states (Arkansas, Idaho, Iowa, Kansas, Minnesota, Missouri, Montana, Nebraska, North Dakota, Oregon, South Dakota, Washington, Wisconsin). Data from Minnesota (1 backyard farm, 99 commercial poultry farms, 1 wild bird) and Iowa (69 commercial poultry farms), where most commercial poultry were affected, predominated in this sample.

To demonstrate the phylogenetic organization of the HPAIV outbreak, we built a maximum-likelihood phylogenetic tree of the concatenated genome (all 8 genome segments) with RAxML version 8.0.0 (*22*) using default parameters and a general time-reversible model with gamma-distributed rate variation among sites. We divided the 265 H5N2 isolates into subgroups of related clusters by their bootstrap value (>70%) on maximum-likelihood phylogenetic analysis (*23*). To assess relatedness support, we performed rapid bootstrapping, with bootstrap convergence criterion yielding 1,000 bootstrap iterations. We illustrated the time series of outbreaks by subgroup and state using Tableau (https://www.tableau.com/).

### Median-Joining Network Analysis

We concatenated all 8 genome segments of each virus isolate to generate a single alignment. We used these alignments to construct a phylogenetic network using the median-joining method implemented in program NETWORK version 5.0 (http://www.fluxus-engineering.com/sharenet_rn.htm) with epsilon set to 0 (*24*).

### Ancestral State Reconstruction of Geographic Location and Host Type

To investigate virus transmission between host types over large spatial scales, we reconstructed the virus transmission history between states geographically using an ancestral state reconstruction approach with a Bayesian stochastic search variable selection to determine the most probable spatial and ecologic transmission history. For all phylogeographic analyses, we used an uncorrelated log-normal distribution relaxed-clock method with a Hasegawa, Kishino, and Yano nucleotide substitution model and Bayesian skyride coalescent prior.

Using a globally derived dataset (n = 127 taxa) of HPAIV hemagglutinin (HA) sequences from Asia, Europe, and North America, we tested whether the HPAIV H5 outbreak in North America resulted from a single virus introduction or multiple virus introductions into the bird population of North America. We then developed a more refined model to estimate viral diffusion and transmission between wild and domestic populations and assess the most likely route of spread. We incorporated HPAIV HA sequences from the US and Canada 2014–2015 outbreaks (*11*) into our discrete phylogenetic model. In this model, we defined geographic region and host type as discrete nominal categories. We categorized geographic region of virus collection by migratory flyway (Pacific, Central, Mississippi) and host type as chicken, turkey, wild bird, or backyard poultry. We estimated ancestral state transition rate and model parameters from a set of 5,000 empirical trees simulated from HPAIV H5 HA gene data collected throughout the outbreaks. For the discrete ancestral state model, we used a nonreversible continuous-time Markov chain model to estimate geographic and host transitions among wild and domestic birds (*25*). We used Bayesian stochastic search variable selection to identify important transitions using a binary indicator (*I*) (*18*,*26*,*27*), enabling the calculation of Bayes factor with SPREAD version 1.0.6 (*28*). A transition was considered important when *I* was >0.5 and Bayes factor was >4.0 (*18*,*26*). We used the last 500 trees from each posterior distribution to construct heat maps representing the average number of transitions per month (Technical Appendix).

## Results

**Figure 1:**
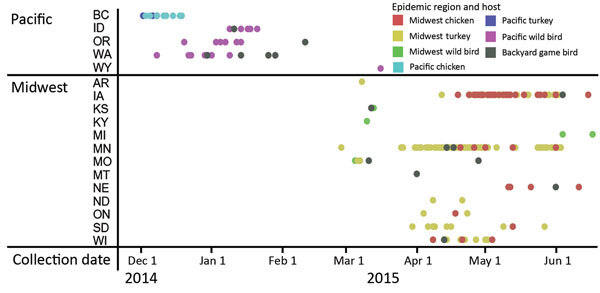
Time series of reassortant highly pathogenic avian influenza virus A(H5N2) distribution, by US state and Canada province, December 2014–June 2015. Virus region and host are indicated. BC, British Columbia, Canada; ON, Ontario, Canada.

The highly pathogenic phenotype of viruses sequenced in this study were confirmed by presence of the polybasic amino acid motif at the HA cleavage site and through experimental infection of chickens by intravenous inoculation (Technical Appendix). Analysis of globally circulating Gs/GD-lineage H5Nx clade 2.3.4.4 viruses showed a widespread distribution and confirmed that the lineage causing the outbreak in North America probably resulted from a single introduction (Technical Appendix Table 2, Figure 1). During December 2014–March 2015, H5N2 was detected in poultry farms of British Columbia and in wild waterfowl and backyard poultry of states along the Pacific flyway (Figure 1). Subsequently, during March–June 2015, H5N2 predominated in poultry outbreaks in the Midwest.

**Figure 2:**
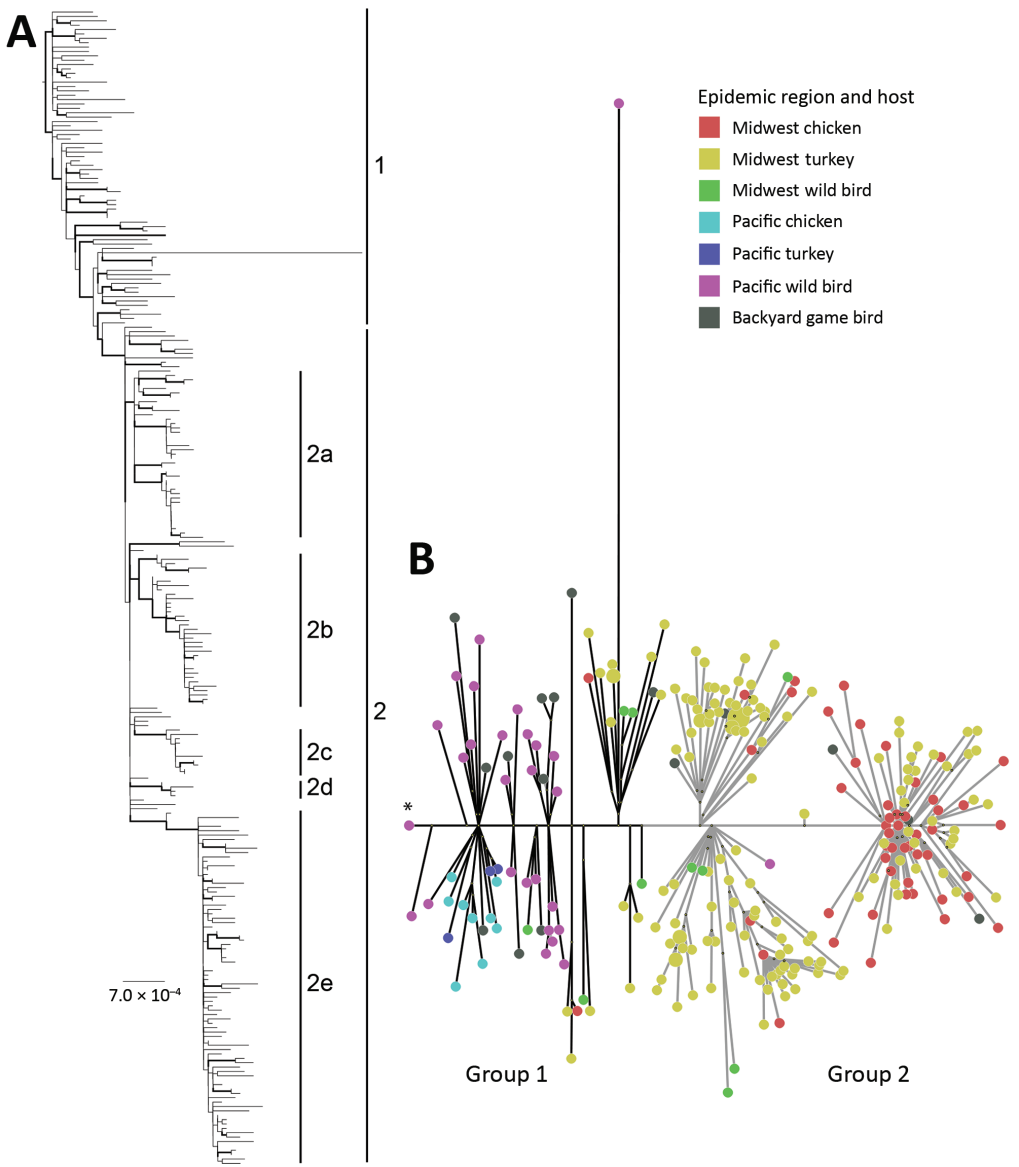
Genetic characterization of reassortant highly pathogenic avian influenza virus A(H5N2) clade 2.3.4.4, North America, 2014–2015. A) Maximum-likelihood phylogeny of concatenated complete genome sequences. Labels indicate genetic subgroups. Scale bar indicates nucleotide substitutions per site. The full version of the phylogenic tree is available in Technical Appendix Figure. B) Median-joining phylogenetic network constructed by using concatenated complete genome sequences. This network includes the most parsimonious trees linking the sequences. Each unique sequence is represented by a circle sized relative to its frequency in the dataset. Virus region and host are indicated. *US index H5N2 virus (A/Northern_pintail/Washington/40964/2014).

We generated a maximum-likelihood phylogenetic tree and median-joining phylogenetic network of HPAIV H5N2 using 265 concatenated full-genome sequences (249 from the United States, 16 from Canada) to investigate the relatedness of isolates (Figure 2; Technical Appendix Figure 2). The H5N2 viruses formed 2 major genetic groups, group 1 and group 2 (includes subgroups 2a, 2b, 2c, 2d, 2e, and 2 nonclustered [2nc]), with maximum-likelihood phylogeny bootstrap proportions >75%. Group 1 contains 73 viruses from 11 US states and 2 Canada provinces across the Pacific (Alaska, Idaho, Montana, Oregon, and Washington, USA; British Columbia), Central (Kansas, South Dakota, and Wyoming, USA), and Mississippi (Arkansas, Minnesota, and Missouri, USA; Ontario, Canada) flyways (Technical Appendix Figures 3, 4). Group 2 predominantly contained viruses isolated from poultry from states along the Mississippi flyway: 2a (n = 39; Iowa, Minnesota, Missouri, Wisconsin); 2b (n = 35; Minnesota); 2c (n = 11; Iowa, Minnesota); 2d (n = 4; Wisconsin); and 2e (n = 81; Iowa, Minnesota; Nebraska, South Dakota in Central flyway). Twenty-two 2nc viruses were detected in 6 states along the Central (North Dakota, South Dakota) and Mississippi (Kentucky, Michigan, Minnesota, Wisconsin) flyways.

Both group 1 and 2 viruses were found in wild birds and gallinaceous poultry (Figure 2; Technical Appendix Figure 3). Wild birds infected with group 2 viruses were only infected with subgroup 2nc viruses. Group 1 viruses were recovered from all host types represented (28 wild birds, 5 raptors, 11 backyard poultry, 14 commercial poultry [1 chicken, 13 turkeys]). Subgroups 2c and 2d were found only in turkeys and had limited geographic spread. The remaining subgroups affected several hosts. Subgroup 2nc viruses were isolated from 6 wild birds, 13 commercial turkey sites, and 3 commercial chicken sites. Subgroup 2a was found in 2 backyard sites, 36 commercial turkey sites, and 1 commercial chicken site. Subgroup 2b predominantly affected commercial turkey sites (n = 32) and 3 commercial chicken flocks; subgroup 2e was recovered from 2 backyard, 36 commercial turkey, and 42 commercial chicken sites and 1 game bird flock. Genetic clustering of subgroups 2a, 2b, and 2e in maximum-likelihood analysis and the close genetic relatedness of Midwest poultry isolates in network analysis provide strong evidence for extensive interfarm transmission (Figure 2; Technical Appendix Figure 2).

**Figure 3:**
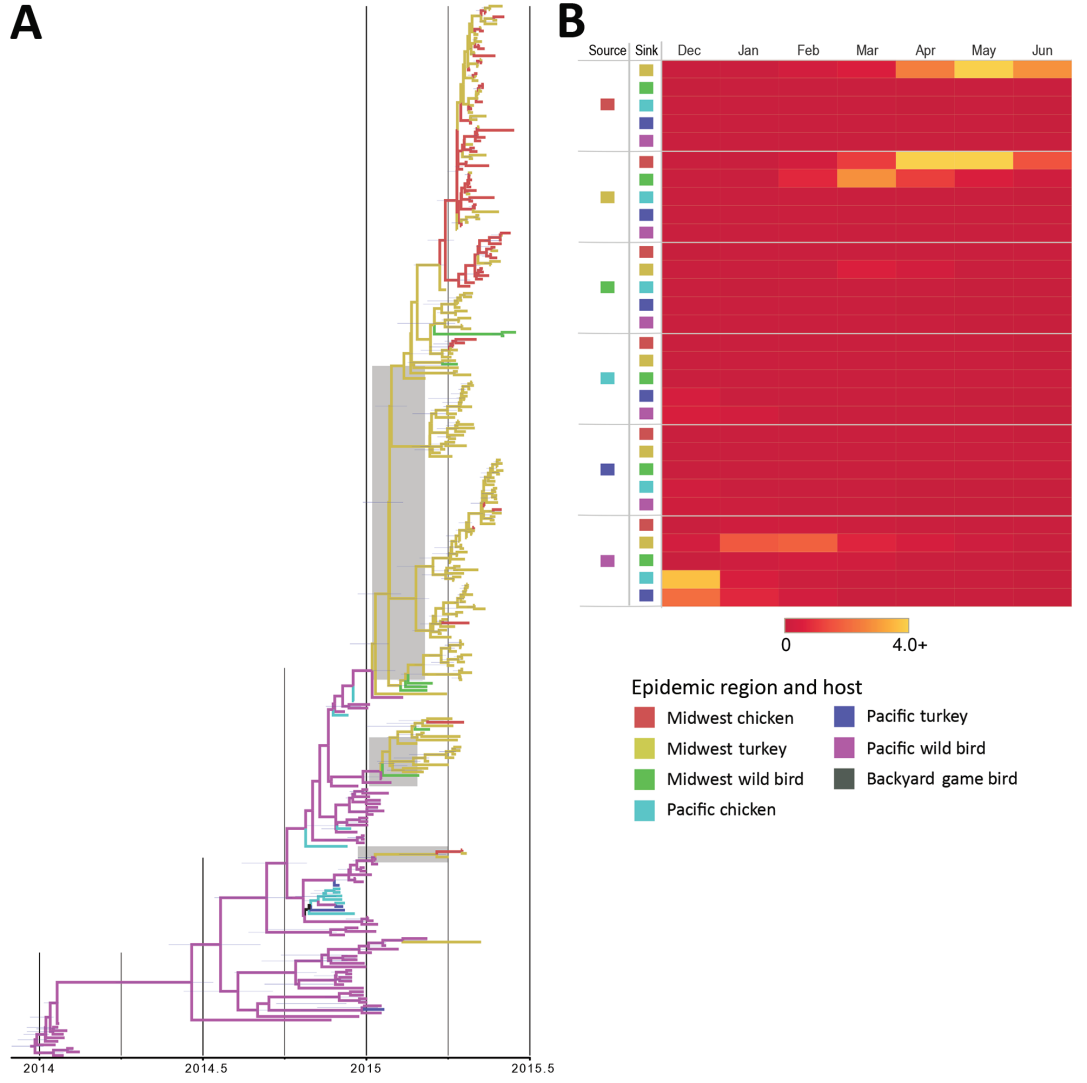
Phylogeographic reconstruction of source–sink dynamics of highly pathogenic avian influenza virus A(H5N2) outbreak, United States, 2014–2015. A) Phylogenetic tree of hemagglutinin gene of H5N2 isolates. The geographic region and host type were defined in the model as discrete nominal character states, and the number of state transitions at tree nodes was counted. The character states included in the phylogenetic model (Midwest chicken, Midwest turkey, Midwest wild bird, Pacific chicken, Pacific turkey, and Pacific wild bird) are indicated. Black nodes and branches represent an ancestral reconstruction that is highly uncertain, sharing equal probability between Pacific wild bird and Pacific turkey. The shaded boxes represent the time between the introduction of the virus into Midwest poultry populations and the first detection of the virus within the population. B) Heat map showing source–sink dynamics and average number of transitions per location per year (0–4.0).

Our phylogenetic analysis suggests that the outbreak in the Midwest was initiated through multiple independent HPAIV H5N2 introductions with group 1 viruses of wild bird origin (Figures 2, 3). To formally test the role of various host populations and elucidate the most likely route for viral spread in the United States, we integrated host type (wild birds, domestic chickens and turkeys, backyard poultry) into our geographic model. Our source–sink and phylogenetic network analyses suggest that the virus detected in the Midwest shares a common ancestor with the viruses detected in the Pacific flyway 1.7–2.4 months before the first detection in Midwest poultry facilities (Figure 3; Technical Appendix Figure 5). Undetected infected hosts also could have migrated from the Pacific Coast through the Midwest, depositing infectious material on the US landscape, leading to the outbreaks in Midwest poultry populations. The virus subsequently rapidly transmitted between commercial turkey and chicken farms. The rapid rate of transmission suggests that underlying epidemiologic factors were driving the spread within and between farm systems.

Bayesian simulation results showed that turkey farms and chicken farms were equally supported as the source of infection of domestic flocks after establishment of the virus in Midwest US farms (Table). Although the discrete state transition rate from chicken farms to turkey farms was higher, this difference is likely an artifact of the substantially larger flocks found in chicken farms, which predominantly use layer houses. Our analysis showed high rates of transmission between turkey and chicken farms in the Midwest, probably confounding control efforts and contributing to the overall extent of the outbreak (Figure 3, panel B). No support exists for viral spread from Midwest wild birds to Midwest commercial farms, suggesting that this epidemiologic pathway likely did not play a role in the spread of the outbreak after the initial introduction into the Midwest (Table), which has further been supported by epidemiologic investigations (*29*). In addition, a transition from Midwest turkey to wild birds is supported by a high Bayes factor (4798.91) and posterior probability (0.999), suggesting HPAIV spillback from domestic poultry to wild birds at a low transition rate (Table).

**Table:** Well-supported discrete trait viral transitions among chicken, turkey, and wild bird populations during highly pathogenic avian influenza virus H5 outbreak, Midwest and Pacific regions, USA, 2014–2015*

Transition from	Transition to	Bayes factor	Posterior probability	Mean transition rate
Midwest chicken	Midwest turkey	38,421.1688	1.0	5.0917
Midwest turkey	Midwest chicken	38,421.1688	1.0	1.8376
Midwest turkey	Midwest wild bird	4,798.9103	0.9991	0.8344
Pacific wild bird	Pacific chicken	464.3334	0.9909	0.8478
Pacific wild bird	Midwest turkey	227.2091	0.9816	0.8557
Pacific wild bird	Pacific turkey	201.2141	0.9792	0.5687

*Only the migrations supported by a Bayes factor >4 and posterior probability >0.5 are indicated.

We identified 14 common substitutions across the entire genome of HPAIV H5N2 when comparing with US index virus A/northern pintail/WA/40964/2014 (H5N2). These substitutions include 10 nonsynonymous substitutions in group 1 and 2 viruses (L386V and V649I in polymerase basic 2; L8F, N130T, and S157P in HA; R253K, E368K, and V412A in neuraminidase; Q78R in matrix 2; I176T in nonstructural protein 1) and 4 nonsynonymous substitutions in group 2 viruses (R215K in polymerase basic 1, A337V in polymerase acidic, N60H in nuclear export protein, K217T in nonstructural protein 1) (Technical Appendix Table 3).

## Discussion

Phylogenetic reconstruction of source–sink dynamics supports multiple independent introductions of HPAIV H5N8 into the United States during late 2014 from wild birds. Despite the frequent detection of HPAIV H5 in wild birds of the Pacific flyway up to February 2015 (*30*), only 2 commercial poultry and 1 backyard farm flocks became infected with HPAIV H5N8 and 7 backyard poultry flocks with HPAIV H5N2 in states along the Pacific flyway. No HPAIV H5N2 infections were found at commercial poultry sites in states along the Pacific flyway (*31*). In contrast with infrequent events in states along the Pacific flyway, 213 H5N2 events in commercial and backyard poultry were reported during March–June 2015 in the Midwest. Our analysis demonstrates extensive interfarm transmission of virus subgroups 2a, 2b, and 2e but not of group 1 viruses, which could also infect wild birds, suggesting a change in the epidemiologic processes driving viral spread. Although a national level–structured surveillance effort was not initiated until July 2015 (*19*), H5N2 detections were far less frequent in the Midwest than in the Pacific flyway, suggesting few H5N2-infected wild waterfowl were in the Central and Mississippi flyways during the outbreak period (*32*,*33*). Nonetheless, detection of H5N2 viruses from wild birds in the Midwest in early 2015 and our phylogenetic analysis suggest that the HPAIV H5N2 moved from the Pacific flyway to the Mississippi and Central flyways as early as January or February 2015, with subsequent interfarm transmission in and between states of the Midwest.

When performing the source–sink phylogenetic analysis, we made assumptions regarding sampling and population structure, which should be considered when interpreting results. For instance, sampling in this analysis is most likely biased toward commercial poultry because of the nature of HPAIV reporting. Oversampling of a specific population might lead to overestimation of that group as a sink to viral migration (*34*); however, a secondary simulation in which viral host characteristics were continuously randomized to observe the influence of sampling heterogeneity (*35*) revealed that sampling does not appear to bias the ancestral reconstruction in this data. Also, in the model, we included the complete random population mixing assumption, even though a structured coalescent approach might have helped limit bias introduced from ecologic barriers that structure wild and domestic bird populations; many of the HPAIV sequences from poultry hosts used in our study represented a single farm. No data were available regarding the size of farms, number of animals infected on those farms, or number of farms affected, all of which might further bias spatial diffusion parameter estimates in a structured coalescent analysis.

The hypothesis that HPAIV H5N2 was introduced into the Midwest through wild birds rather than poultry production channels is supported by epidemiologic evidence; during February 27–April 20, 2015, H5N2 virus was detected 17 times across 5 states and 16 counties in different hosts (wild bird, raptor, backyard flocks, turkey flocks), and epidemiologic links could not be found between Pacific Coast and Midwest farms for any virus subgroup. The widespread detection of the HPAIV H5 lineage in healthy wild birds (*30*), subclinical infection with high virus shedding, and transmission among mallard ducks experimentally infected with HPAIV H5N2 (*36*) support the hypothesis of HPAIV H5 dissemination to multiple states through wild waterfowl over a short period during wild aquatic bird migrations. For the outbreak in the Midwest (February–June 2015), events (infections with viruses of groups 1 and 2nc) detected early during the outbreak (February–April 2015) probably represent independent introductions, followed by limited secondary spread, and events (infections with viruses of subgroups 2a–e) detected later during the outbreak (April–June 2015) were largely caused by extensive interfarm transmission. Previous estimates of basic reproductive number and viral migration among host types in domestic and wild birds support our findings that HPAIV H5N2 rapidly spread between poultry facilities in the Midwest after the initial introduction of a virus of wild bird origin (*37*). Grear et al. showed that the H5N2 and H5N8 HPAIV outbreak did not encounter sufficient transmission barriers to prevent persistence when introduced to wild or domestic birds (*37*). Consistent with our data, Grear et al. concluded that once the HPAIV H5N2 entered the poultry production system in the Midwest, transmission was driven through poultry production–related mechanisms (*37*). That conclusion was based on evidence of a close phylogenetic distance among sequences from poultry facilities, relatively infrequent cross-species transmission, and a high estimated proportion of virus diversity in addition to the lack of detection of this lineage in reservoir hosts when using other surveillance data. Our Bayesian simulation also supports the high probability of 2-way transmission between poultry and wild bird populations.

The epidemiology of the HPAIV H5 detections and pathobiologic features suggest that the early H5N8 and H5N2 group 1 viruses detected in the United States were highly adapted to waterfowl and poorly adapted to chickens and turkeys (*36*,*38*–*40*). Index wild bird viruses [A/gyrfalcon/Washington/40188–6/2014 (H5N8), A/northern pintail/Washington/40964/2014 (H5N2)] required a high mean bird infectious dose of 50% (BID_50_; 10^5.7^ EID_50_) to infect chickens; directly inoculated and in-contact exposed survivors did not seroconvert (*38*). Increased virus adaptation to chickens and turkeys, specifically poultry, was observed among the H5N2 group 2 viruses and not the H5N2 group 1 viruses (*39*,*40*). In chickens, the BID_50_ of H5N2 group 2 viruses for poultry (10^3.2–3.6^ EID_50_) was lower than that of group 1 viruses for poultry (10^5.1^ EID_50_) and index wild birds (10^5.7^ EID_50_).

Examination of mutational frequencies and patterns showed common mutations among H5N2 subgroup 2a–e viruses fixed in the population during circulation in poultry. In particular, substitutions in the nucleoprotein amino acid 105 (M105V, M105I) were identified among H5N2 group 2 viruses from the United States and a virus isolate from a chicken in British Columbia (*11*) and has been suggested as a determinant for virus adaptation from ducks to chickens (*41*). Consistent with our previous study (*39*), we identified common substitutions (R215K polymerase basic 1, A337V polymerase acidic, N60H nuclear export protein, K217T nonstructural protein 1) in H5N2 group 2 viruses that increased infectivity and pathogenicity in chickens in comparison with group 1 viruses. In addition, we found substitution S157P at an antigenic site of the HA protein, which might affect the immunogenicity profile of the virus (*42*).

In summary, we analyzed the transmission dynamics of Asia-origin HPAIV H5 in the United States. The H5N8 virus came from East Asia, entered North America during the fall 2014 migration season (*7*), and spread rapidly along the wild bird flyways in the United States starting in December 2014. In the Pacific flyway, Eurasia H5N8 virus circulated in wild birds and disseminated to backyard and commercial poultry farms largely by point-source introductions; a single detection occurred in a backyard flock in Indiana outside the Pacific states. Furthermore, influenza A genome segments of the Eurasia H5N8 virus mixed with segments of the North America low pathogenicity avian influenza viruses, creating new reassortant HPAIVs H5N1, H5N2, and H5N8. For the reassortant H5N2 virus in the Midwest, our results strongly support introduction from the Pacific flyway early in 2015, with >3 initial independent introductions and late events involving extensive secondary spread between poultry farms. These H5N8 and H5N2 viruses cause substantial illness and death in poultry but have, thus far, not been implicated in human infection. Mammalian studies in mice and ferrets suggest low public health risk (*43*,*44*).

During 2016–2017, HPAIV H5N8 clade 2.3.4.4 group B (Gochang-like) reemerged and caused outbreaks in wild birds and domestic poultry across Europe, Asia, and Africa (*45*,*46*). Enhanced biosecurity is required to prevent the introduction and dissemination of HPAIVs across the poultry industry; the role that human activity can play in viral spread via fomites should not be underestimated. Continued surveillance, virus characterization, and infectivity studies remain invaluable to monitoring the spread and evolution of these H5 viruses; such efforts could further the design of improved prevention strategies. Additional research is needed to decipher the potential mechanisms of virus introduction into poultry systems.

Technical Appendix Additional details on analysis of highly pathogenic avian influenza virus (HPAIV) A(H5Nx) clade 2.3.4.4 isolates from 2014–2015 outbreak in North America.
