# Supplementary material for: Transmission Dynamics of Highly Pathogenic Avian Influenza Virus A(H5Nx) Clade 2.3.4.4, North America, 2014–2015
Source: Emerg Infect Dis. 2018 Oct;24(10):1840–8. doi: 10.3201/eid2410.171891 (PMC6154162; doi:10.3201/eid2410.171891)
Supplement: Technical Appendix — Additional details on analysis of highly pathogenic avian influenza virus (HPAIV) A(H5Nx) clade 2.3.4.4 isolates from 2014–2015 outbreak in North America. [file 17-1891-Techapp-s1.pdf]

Article DOI: https://doi.org/10.3201/eid2410.171891

Transmission Dynamics of Highly Pathogenic Avian Influenza Virus A(H5Nx) Clade 2.3.4.4, North America, 2014–2015

Technical Appendix

Ancestral State Reconstruction of the Geographic Location and Host Type

To investigate the viral transmission between host types over large spatial scales, we reconstructed the viral transmission history between geographic states using an ancestral state reconstruction approach with a Bayesian stochastic search variable selection to determine the most probable spatial and ecologic transmission history. For all phylogeographic analyses, we used an uncorrelated lognormal distribution relaxed-clock method with a Hasegawa, Kishino, and Yano nucleotide substitution model and the Bayesian skyride coalescent prior. A Markov chain Monte Carlo method to sample trees and evolutionary parameters was run for 7.0×10^7 generations. At least 3 independent Markov chains were performed to achieve output that converged well, on the basis of the criteria of an effective sampling size of >200 as calculated by Tracer version 1.5 with a 10% burn-in (http://tree.bio.ed.ac.uk/software/tracer/). A maximum clade credibility tree was generated for each data set by using TreeAnnotator (http://www.phylo.org/index.php/tools/treeannotator.html) in BEAST (http://beast.community/). FigTree 1.3.1 (http://tree.bio.ed.ac.uk/) was used for visualization of trees.

Using a globally derived data set (ntax = 127), we tested whether the highly pathogenic avian influenza virus (HPAIV) H5 that caused the North America outbreak was the result of a single introduction or multiple introductions into the North America wild bird population using hemagglutinin (HA) sequences of HPAIVs from Asia, Europe, and North America. We then developed a more refined model to estimate viral diffusion and transmission between wild and domestic populations and assess the most likely route for spread. Sequences of the HPAIV HAs from the United States and Canada (*1*) during the 2014–15 outbreak and the 12 most closely related H5N8 sequences from Asia (determined from our global scale analysis), which were

collected in 2014, were incorporated into our analysis discrete phylogenetic model. The geographic region and host type were defined in the model as discrete nominal categories. The geographic region of collection was categorized by migration flyway (Pacific, Central, Mississippi). No H5Nx viruses were detected in US poultry from the Atlantic flyway region, and there was no US Department of Agriculture Wildlife Services wild bird surveillance conducted in the Atlantic flyway during the period of interest. Sequences of viruses from the Central and Mississippi flyways were combined into 1 category due to the sparsity of sequences from the Central flyway. Host type was categorized into chicken, turkey, wild bird, and backyard poultry. We assumed that backyard poultry operations were small-scale operations with lower biosecurity than large-scale commercial poultry production. We, therefore, treated the backyard poultry category as ambiguous state, which is a general class accounting for various-sized backyard operations. We estimated the ancestral state transition rate and model parameters from a set of 5,000 empirical trees simulated using HPAIV H5 HA gene data collected throughout the outbreak. We assumed a constant coalescent tree before produce the empirical phylogenetic tree set. For the discrete ancestral state model, we used a nonreversible continuous-time Markov chain model with a strict clock assumption to estimate geographic and host transitions among wild and domestic birds in the US outbreak during December 2014–June 2015 (*2*). Bayesian stochastic search variable selection was used to identify important transitions by using a binary indicator (*I*) (*3–5*), enabling the calculation of Bayes factors with SPREAD version 1.0.6 (*6*). A transition was considered important when $I > 0.5$ and Bayes factors >4.0 (*3*,*4*). At least 3 independent runs of Markov chains were performed to achieve output that converged well. The last 500 trees from each posterior distribution of trees were used to construct heat maps representing the average number of transitions per month. Convergence of tree topologies was assessed by using RWTY, an R implementation of Are We There Yet (*7*).

Intravenous Pathogenicity Index in Chickens

The intravenous pathogenicity index (IVPI) test of 20 H5 isolates, representing at least the index cases for each state and domestic species, was conducted according to the World Organisation for Animal Health Manual of Diagnostic Tests and Vaccines for Terrestrial Animals (http://www.oie.int/en/international-standard-setting/terrestrial-manual). In brief, 0.1 mL of infectious allantoic fluid was inoculated intravenously into ten 6-week-old specific–

pathogen-free chickens and chickens monitored for 10 days for morbidity and mortality. Isolates with IVPI >1.2 were characterized as HPAIV. The challenge studies and all experiments with live viruses were conducted in a biosafety level 3 facility at the National Veterinary Services Laboratories, Animal and Plant Health Inspection Service, US Department of Agriculture in Ames, Iowa, USA, and in accordance with approved institutional animal care and use protocols. Twenty representative isolates were highly pathogenic in chickens on intravenous inoculation and IVPIs of each isolate ranged 2.57–2.99 (Technical Appendix Table 1).

Single-Nucleotide Polymorphism (SNP) Analysis for HPAIV H5N2

To identify genetic changes, full-genome sequence analysis of the US HPAIV H5N2 isolates was conducted. For phylogenetic analysis, complete coding regions of the US HPAIV H5 isolates were aligned by using MAFFT (https://mafft.cbrc.jp/alignment/software/) and were used for subsequent SNP testing. The coding sequences discriminating SNPs were classified as either nonsynonymous or synonymous depending on whether or not they corresponded to differences in encoded amino acid sequences. Nonsynonymous substitutions found in the alignment of 264 H5N2 complete genome sequences from the United States and Canada were compared with A/Northern pintail/Washington/40964/2014 (H5N2) virus, previously identified as the oldest US H5 2.3.4.4 representative at 5% of minimum variant frequency.

A total of 31 nonsynonymous substitutions across the entire genome were identified in HPAIV H5N2 when compared with the US index virus A/Northern Pintail/WA/ 2014 (Technical Appendix Table 3). A total of 10 nonsynonymous substitutions were identified in group 1 viruses and maintained in group 2 H5N2 viruses: L386V and V649I in polymerase basic 2; L8F, N130T, and S157P in HA; R253K, E368K, and V412A in neuraminidase; Q78R in matrix 2; and I176T in nonstructural protein 1. The nonsynonymous substitutions R215K in polymerase basic 1, A337V in polymerase acidic, P152S in HA, N60H in nuclear export protein, and K217T in nonstructural protein 1 were common substitutions of H5N2 group 2 viruses. The other 16 nonsynonymous substitutions were unique changes identified from each subgroup.

Transmission Pattern of H5N8 HPAIV in the United States

The 8 gene segments were concatenated to generate a single alignment that was used to construct a phylogenetic network by using the median-joining method implemented in the program NETWORK version 5.0 with epsilon set to 0 (*8*).

Phylogenetic network analysis of the HPAIV H5N8 suggests largely independent introductions with limited lateral spread (Technical Appendix Figure 6). There was a single detection of H5N8 outside the Pacific flyway in a backyard flock in Indiana. The phylogenetic reconstruction of source–sink dynamics supports the potential for wild bird introduction in Indiana (main text Figure 3; Technical Appendix Figure 5); however, there were no H5N8 detections in wild birds outside the Pacific flyway.

References

1. Xu W, Berhane Y, Dubé C, Liang B, Pasick J, VanDomselaar G, et al. Epidemiological and evolutionary inference of the transmission network of the 2014 highly pathogenic avian influenza h5n2 outbreak in British Columbia, Canada. Sci Rep. 2016;6:30858. PubMed http://dx.doi.org/10.1038/srep30858

2. Minin VN, Suchard MA. Counting labeled transitions in continuous-time Markov models of evolution. J Math Biol. 2008;56:391–412. PubMed http://dx.doi.org/10.1007/s00285-007-0120-8

3. Bahl J, Krauss S, Kühnert D, Fourment M, Raven G, Pryor SP, et al. Influenza A virus migration and persistence in North American wild birds. PLoS Pathog. 2013;9:e1003570. PubMed http://dx.doi.org/10.1371/journal.ppat.1003570

4. Bahl J, Pham TT, Hill NJ, Hussein IT, Ma EJ, Easterday BC, et al. Ecosystem interactions underlie the spread of avian influenza A viruses with pandemic potential. PLoS Pathog. 2016;12:e1005620. PubMed http://dx.doi.org/10.1371/journal.ppat.1005620

5. Lemey P, Rambaut A, Drummond AJ, Suchard MA. Bayesian phylogeography finds its roots. PLOS Comput Biol. 2009;5:e1000520. PubMed http://dx.doi.org/10.1371/journal.pcbi.1000520

6. Bielejec F, Rambaut A, Suchard MA, Lemey P. SPREAD: spatial phylogenetic reconstruction of evolutionary dynamics. Bioinformatics. 2011;27:2910–2. PubMed http://dx.doi.org/10.1093/bioinformatics/btr481

7. Warren DL, Geneva AJ, Lanfear R. RWTY (R We There Yet): an R package for examining convergence of Bayesian phylogenetic analyses. Mol Biol Evol. 2017;34:1016–20. PubMed

8. Bandelt HJ, Forster P, Röhl A. Median-joining networks for inferring intraspecific phylogenies. Mol Biol Evol. 1999;16:37–48. PubMed http://dx.doi.org/10.1093/oxfordjournals.molbev.a026036

Technical Appendix Table 1. Pathogenicity of highly pathogenic avian influenza viruses H5Nx in chickens during outbreaks, United States, 2014–15

Strain name	Date collected	IVPI index
A/gyrfalcon/Washington/41088–6/2014(H5N8)	2014 Dec 7	2.65
A/Northern_pintail/Washington/40964/2014(H5N2)	2014 Dec 8	2.57
A/guinea_fowl/Washington/41613–1/2014(H5N8)	2014 Dec 16	2.69
A/Coopers hawk/Washington/197127/2014(H5N2)	2014 Dec 29	2.77
A/American_green-winged_teal/Washington/195750/2014(H5N1)	2014 Dec 29	2.99
A/chicken/Washington/61–9/2014(H5N2)	2014 Dec 30	2.87
A/chicken/Idaho/1711–7/2015(H5N2)	2015 Jan 11	2.70
A/turkey/California/K1500169–1.2/2015(H5N8)	2015 Jan 21	2.75
A/chicken/California/4912/2015(H5N8)	2015 Feb 6	2.96
A/chicken/Oregon/5256/2015(H5N2)	2015 Feb 11	2.67
A/turkey/Minnesota/7172–1/2015(H5N2)	2015 Feb 27	2.80
A/turkey/Missouri/15–007478–1/2015(H5N2)	2015 Mar 6	2.91
A/turkey/Arkansas/7791–1/2015(H5N2)	2015 Mar 8	2.81
A/chicken/Kansas/8395–3/2015(H5N2)	2015 Mar 12	2.66
A/turkey/South Dakota/10371/2015(H5N2)	2015 Mar 30	2.86
A/chicken/Montana/10559/2015(H5N2)	2015 Apr 1	2.74
A/chicken/Wisconsin/11595–1/2015(H5N2)	2015 Apr 8	2.94
A/turkey/North Dakota/15–011419–1/2015(H5N2)	2015 Apr 8	2.96
A/turkey/Iowa/11762–1/2015(H5N2)	2015 Apr 12	2.92
A/chicken/Indiana/15–014961–4/2015(H5N8)	2015 May 9	2.80

IVPI, intravenous pathogenicity index.

Technical Appendix Table 2. Migration of viruses from Asia well-supported by Bayes factors*

Migrations	Bayes factor	Posterior probability	Mean migration rate
China→Vietnam	73.343	0.9096	0.584
Japan→Taiwan	56.543	0.8859	1.352
China→Korea	42.831	0.8546	0.806
China→Laos	40.668	0.8481	0.589
Korea→Japan	35.5599	0.8300	2.563
Japan→North America	11.6459	0.6152	1.268
Japan→Europe	11.4887	0.6120	1.166

*Migrations supported by a Bayes factor >6 and posterior probability >0.5.

Technical Appendix Table 3. Nonsynonymous substitutions found in highly pathogenic avian influenza virus H5N2 isolates from United States outbreaks when compared with A/northern pintail/Washington/40964/2014 (H5N2), 2014–15*

Protein	Substitution	Codon change	Group or subgroup
PB2	K54R	AAA→AGA	2e
PB2	L386V	TTA→GTA	1, 2a, 2b, 2c, 2d, 2e
PB2	V649I	GTA→ATA	1, 2a, 2b, 2c, 2d, 2e
PB1	E180D	GAA→GAC	2e
PB1	R215K	AGG→AAG	2a, 2b, 2c, 2d, 2e
PB1	M317V	ATG→GTG	2e
PB1	R723L	CGA→CTA	2a
PA	A337V	GCT→GTT	2a, 2b, 2c, 2d, 2e
PA	A475T	GCA→ACA	2e
HA	L6I	CTT→ATT	2c
HA	L7P	CTT→CCT	2e
HA	L8F	CTT→TTT	1, 2a, 2b, 2c, 2d, 2e
HA	M82I	ATG→ATA	2e
HA	N130T	AAT→ACT	1, 2a, 2b, 2c, 2d, 2e
HA	P152S	CCA→TCA	2a, 2b
HA	S157P	TCC→CCC	1, 2a, 2b, 2c, 2d, 2e
HA	K250R	AAA→AGA	2a
HA	L338Q	CTA→CAA	2e
NP	M105V	ATG→GTG	2b

Protein	Substitution	Codon change	Group or subgroup
NP	M105I	ATG→ATA	2a
NP	I109T	ATC→ACC	2e
NA	R253K	AGA→AAA	1, 2a, 2b, 2c, 2d, 2e
NA	E368K	GAA→AAA	1, 2a, 2b, 2c, 2d, 2e
NA	T380I	ACC→ATC	2b
NA	V412A	GTT→GCT	1, 2a, 2b, 2c, 2d
NA	S416G	AGC→GGC	2e
NA	R430K	AGG→AAG	2b
M2	Q78R	CAG→CGG	1, 2a, 2b, 2c, 2d, 2e
NEP	N60H	AAC→CAC	2a, 2b, 2c, 2d, 2e
NS1	I176T	ATT→ACT	1, 2a, 2b, 2c, 2d, 2e
NS1	K217T	AAA→ACA	2a, 2b, 2c, 2d, 2e

*HA, hemagglutinin; M2, matrix 2; NA, neuraminidase; NEP, nuclear export protein; NP, nucleoprotein; NS1, nonstructural protein 1; PA, polymerase acidic; PB1, polymerase basic 1; PB2, polymerase basic 2.

Technical Appendix Table 4. Acknowledgment of GISAID EpiFlu submitters*

Segment ID	Isolate name	Collection date	Submitter	Originating laboratory
EPI169427	A/wigeon/Sakha/1/2014	2014 Sep 25	Ivan Susloparov	State Research Center of Virology and Biotechnology Vector
EPI161973	A/environment/Shenzhen/25–24/2013	2013 Dec 2	Guang Liu	Beijing Genomics Institute Shenzhen
EPI137255	A/duck/Hebei/3/2011	2011 Dec 7	Di Liu	Institute of Microbiology, Chinese Academy of Sciences
EPI137254	A/duck/Hebei/2/2011	2011 Dec 1	Di Liu	Institute of Microbiology, Chinese Academy of Sciences
EPI163493	A/Sichuan/26221/2014	2014 Apr 21	Lei Yang	WHO Chinese National Influenza Center
EPI161972	A/duck/Jiangxi/95/2014	2014 Jan 10	Guang Liu	Beijing Genomics Institute Shenzhen
EPI169424	A/chicken/Miyazaki/7/2014	2014 Dec 16	Takehiko Saito	National Institute of Animal Health
EPI169390	A/crane/Kagoshima/KU1/2014	2014 Nov 23	Makoto Ozawa	Kagoshima University
EPI168075	A/chicken/Netherlands/14015531/2014	2014 Nov 15	Guus Koch	Central Veterinary Institute
EPI168025	A/duck/Chiba/26–372–61/2014	2014 Nov 18	Takehiko Saito	National Institute of Animal Health
EPI168024	A/duck/Chiba/26–372–48/2014	2014 Nov 18	Takehiko Saito	National Institute of Animal Health
EPI167905	A/Chicken/Netherlands/14015526/2014	2014 Nov 14	Guus Koch	Central Veterinary Institute
EPI167904	A/duck/England/36254/14	2014 Nov 14	Amanda Hanna	Animal and Plant Health Agency
EPI166694	A/duck/Beijing/CT01/2014	2014 Jan 22	Di Liu	Institute of Microbiology, Chinese Academy of Sciences
EPI166693	A/duck/Beijing/FS01/2014	2014 Jan 20	Di Liu	Institute of Microbiology, Chinese Academy of Sciences
EPI159719	A/Chicken/Kumamoto/1–7/2014	2014 Apr 13	Michiyo Yoshizawa Harada	National Institute of Animal Health
EPI173568	A/green-winged teal/Korea/KU-12/2015	2015 Jan 22	Dong-Hun Lee	Konkuk University
EPI169273	A/turkey/Germany/R2474-L00899/2014	2014 Nov 4	Anne Pohlmann	Friedrich-Loeffler-Institut
EPI586509	A/poultry/BC/FAV19/2014	2014 Dec 8	CFIA	Animal Health Centre, Ministry of Agriculture
EPI584079	A/turkey/BC/FAV14/2014	2014 Dec 6	CFIA	Animal Health Centre, Ministry of Agriculture
EPI586501	A/poultry/BC/FAV17/2014	2014 Dec 5	CFIA	Animal Health Centre, Ministry of Agriculture
EPI586493	A/poultry/BC/FAV15/2014	2014 Dec 5	CFIA	Animal Health Centre, Ministry of Agriculture
EPI586550	A/chicken/BC/FAV24/2014	2014 Dec 18	CFIA	Animal Health Centre, Ministry of Agriculture
EPI586542	A/chicken/BC/FAV23/2014	2014 Dec 15	CFIA	Animal Health Centre, Ministry of Agriculture
EPI586534	A/chicken/BC/FAV22/2014	2014 Dec 12	CFIA	Animal Health Centre, Ministry of Agriculture
EPI586526	A/chicken/BC/FAV21/2014	2014 Dec 11	CFIA	Animal Health Centre, Ministry of Agriculture
EPI586518	A/chicken/BC/FAV20/2014	2014 Dec 8	CFIA	Animal Health Centre, Ministry of Agriculture
EPI586558	A/chicken/BC/FAV25/2014	2014 Dec 19	CFIA	Animal Health Centre, Ministry of Agriculture

*CFIA, Canadian Food Inspection Agency; GISAID, global initiative on sharing all influenza data; ID, identification; WHO, World Health Organization.

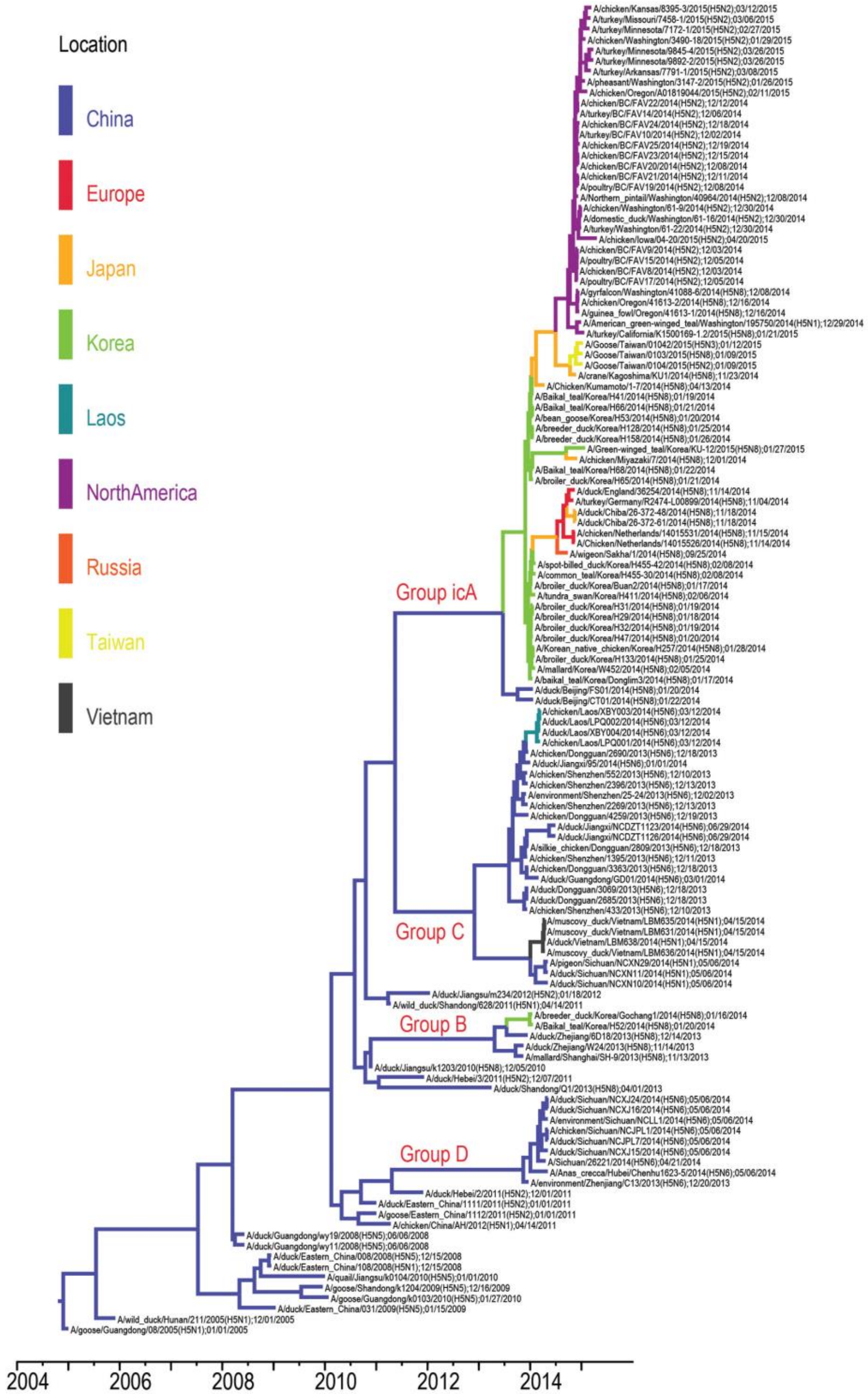

Technical Appendix Figure 1. Phylogeography of hemagglutinin of highly pathogenic avian influenza virus H5 clade 2.3.4.4 isolates from outbreaks, North America, 2014–2015. The phylogenetic relationships and temporal evolutionary history were estimated by molecular clock analysis. Group designations are indicated. Scale bar indicates year of isolation.

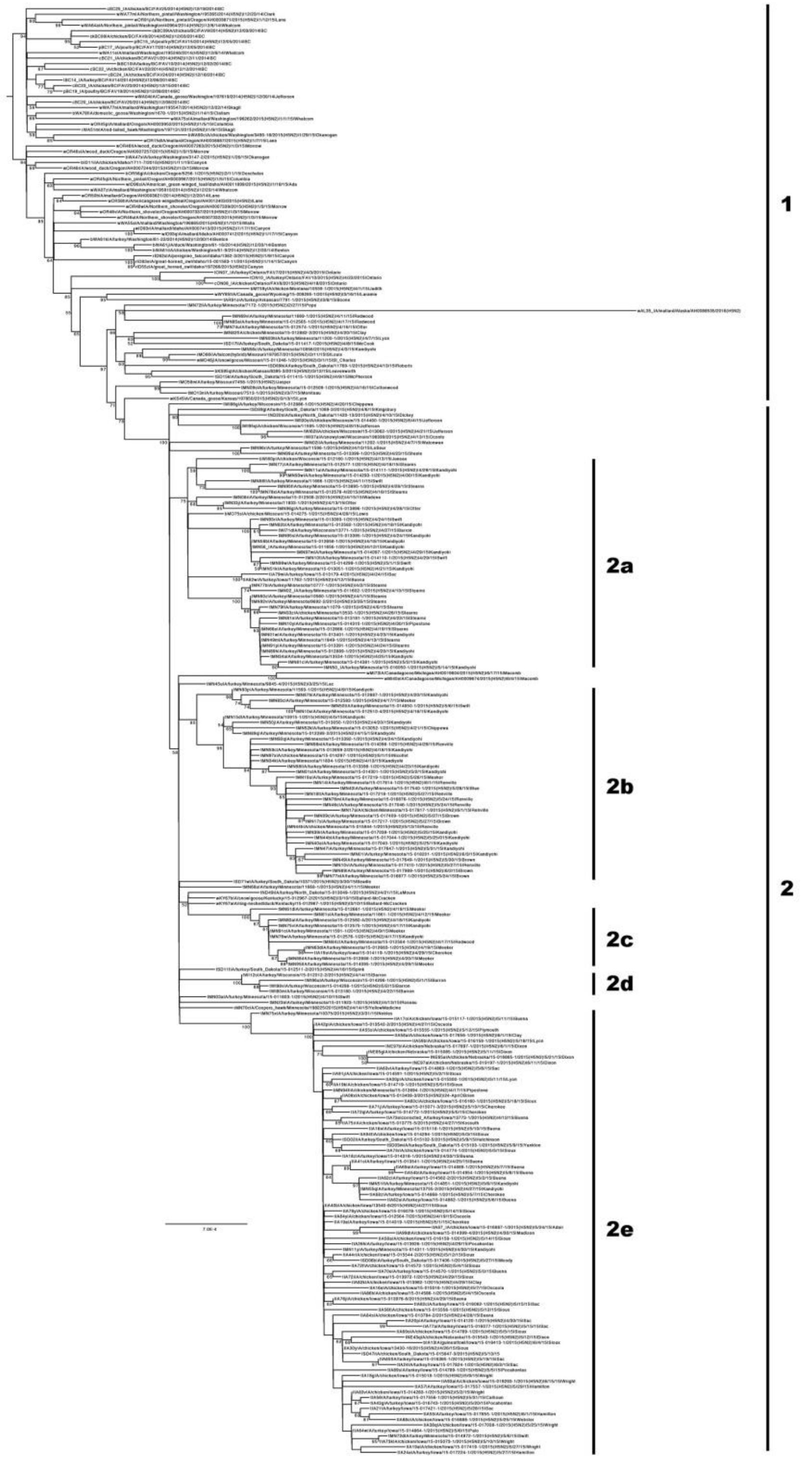

Technical Appendix Figure 2. Maximum-likelihood phylogeny of concatenated complete genome sequences of highly pathogenic avian influenza virus H5N2 clade 2.3.4.4, North America, 2014–2015. Strain names are displayed. Numbers along branches indicate bootstrap values >70%. Brackets indicate the genetic subgroups. Scale bar indicates nucleotide substitutions per site.

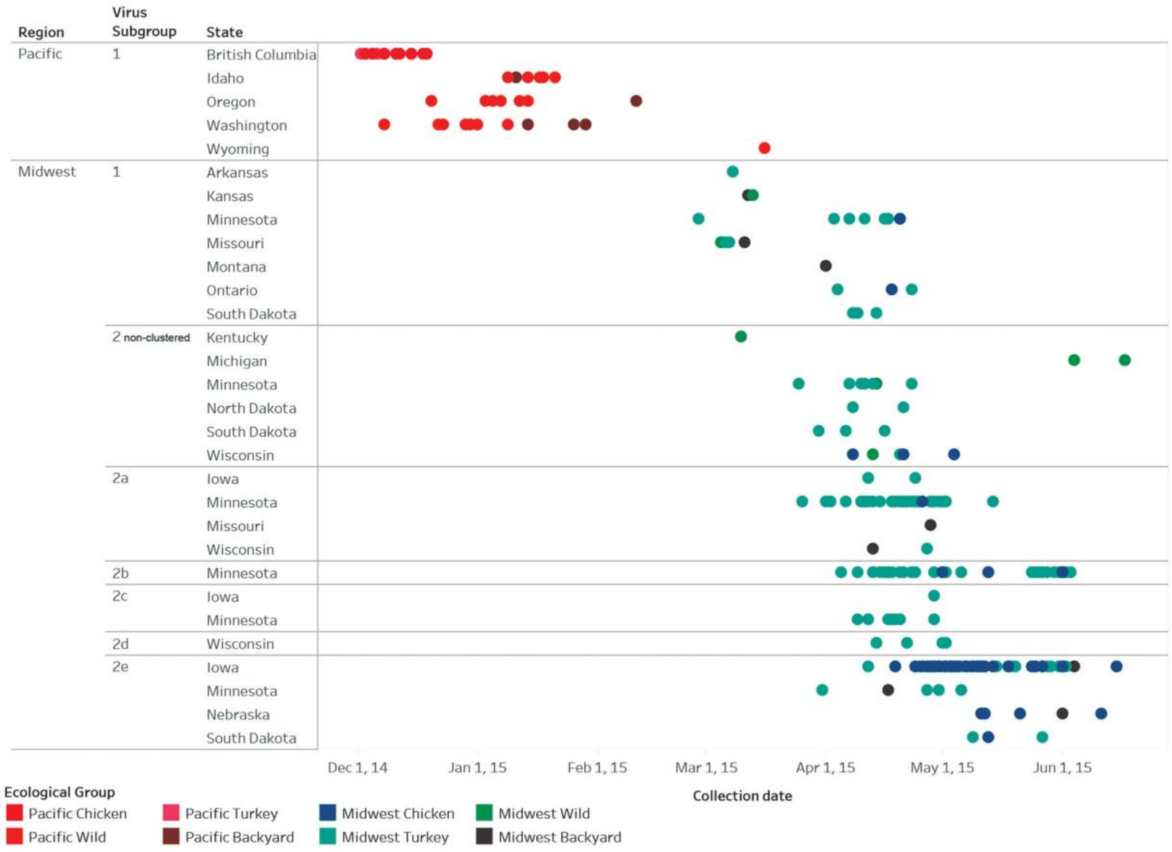

Technical Appendix Figure 3. Time series of highly pathogenic avian influenza virus H5N2 distribution during outbreaks by subgroup and US state or Canada province of North America, 2014–2015. Ecologic group is indicated.

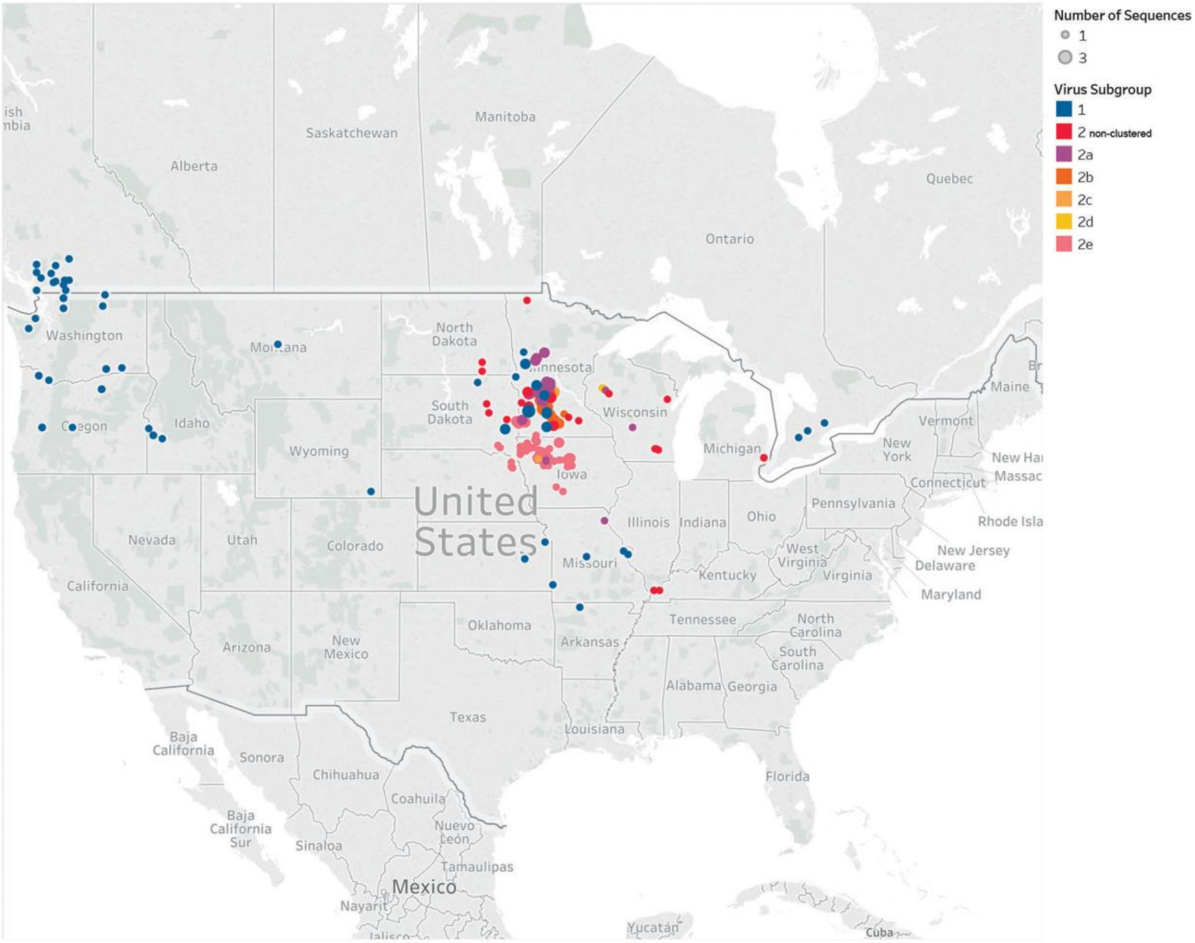

Technical Appendix Figure 4. Distribution of highly pathogenic avian influenza virus H5N2 subgroup during outbreak, North America, 2014–2015. Virus subgroup is indicated.

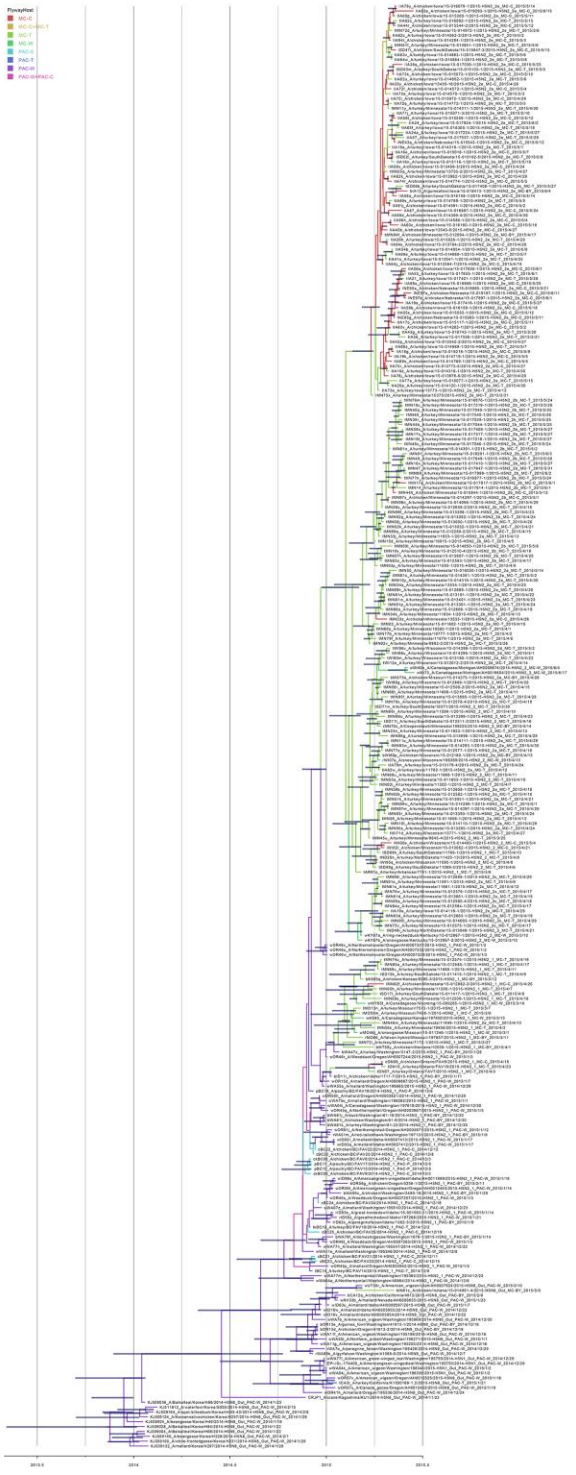

Technical Appendix Figure 5. Bayesian phylogenetic analysis and ancestral state reconstruction of highly pathogenic avian influenza virus H5 hemagglutinin gene, North America. The outgroup contains avian influenza viruses from Asia. Color changes at nodes represent transition between discrete character states (a combination of geographic region and host type). Bars represent the node height 95% highest posterior density. C, chicken; MC, Mississippi-Central; PAC, Pacific; T, turkey; W, wild bird.

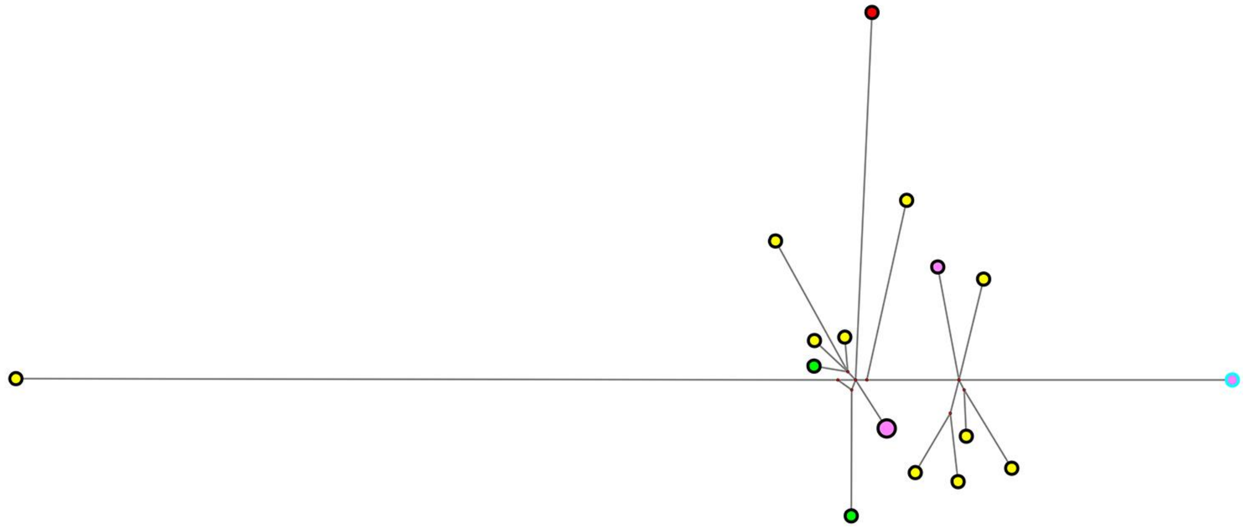

Technical Appendix Figure 6. Median-joining phylogenetic network of highly pathogenic avian influenza H5N8 viruses, North America, 2014–2015. The network was constructed from the concatenated entire genome of H5N8 viruses and includes all the most parsimonious trees linking the sequences. Each unique sequence is represented by a circle sized relative to its frequency in the data set. Circles were colored according to sample origin: red is commercial poultry, purple backyard poultry, yellow wild birds, and green raptors. Outer circle color indicates flyway: black Pacific and sky-blue Mississippi-Central.
